# Sequence Decision Transformer for Adaptive Traffic Signal Control

**DOI:** 10.3390/s24196202

**Published:** 2024-09-25

**Authors:** Rui Zhao, Haofeng Hu, Yun Li, Yuze Fan, Fei Gao, Zhenhai Gao

**Affiliations:** 1 College of Automotive Engineering, Jilin University, Changchun 130025, China; rzhao@jlu.edu.cn (R.Z.); huhf24@mails.jlu.edu.cn (H.H.); fanyz23@mails.jlu.edu.cn (Y.F.); 2 Graduate School of Information and Science Technology, The University of Tokyo, Tokyo 113-8654, Japan; li-yun@g.ecc.u-tokyo.ac.jp; 3 National Key Laboratory of Automotive Chassis Integration and Bionics, Jilin University, Changchun 130025, China; gaozh@jlu.edu.cn

**Keywords:** adaptive traffic signal control, deep reinforcement learning, Markov decision process, attention mechanism, proximal policy optimization

## Abstract

Urban traffic congestion poses significant economic and environmental challenges worldwide. To mitigate these issues, Adaptive Traffic Signal Control (ATSC) has emerged as a promising solution. Recent advancements in deep reinforcement learning (DRL) have further enhanced ATSC’s capabilities. This paper introduces a novel DRL-based ATSC approach named the Sequence Decision Transformer (SDT), employing DRL enhanced with attention mechanisms and leveraging the robust capabilities of sequence decision models, akin to those used in advanced natural language processing, adapted here to tackle the complexities of urban traffic management. Firstly, the ATSC problem is modeled as a Markov Decision Process (MDP), with the observation space, action space, and reward function carefully defined. Subsequently, we propose SDT, specifically tailored to solve the MDP problem. The SDT model uses a transformer-based architecture with an encoder and decoder in an actor–critic structure. The encoder processes observations and outputs, both encoded data for the decoder, and value estimates for parameter updates. The decoder, as the policy network, outputs the agent’s actions. Proximal Policy Optimization (PPO) is used to update the policy network based on historical data, enhancing decision-making in ATSC. This approach significantly reduces training times, effectively manages larger observation spaces, captures dynamic changes in traffic conditions more accurately, and enhances traffic throughput. Finally, the SDT model is trained and evaluated in synthetic scenarios by comparing the number of vehicles, average speed, and queue length against three baselines, including PPO, a DQN tailored for ATSC, and FRAP, a state-of-the-art ATSC algorithm. SDT shows improvements of 26.8%, 150%, and 21.7% over traditional ATSC algorithms, and 18%, 30%, and 15.6% over the FRAP. This research underscores the potential of integrating Large Language Models (LLMs) with DRL for traffic management, offering a promising solution to urban congestion.

## 1. Introduction

Urban traffic congestion has long been a global issue, negatively impacting both the economy and the environment. The energy used during instances of traffic congestion contributes to the emission of greenhouse gases such as carbon dioxide, exacerbating the greenhouse effect. In the United States, traffic congestion in just one year has been estimated to result in economic damages totaling USD 121 million, along with the generation of 25,396 tons of carbon dioxide [1]. Meanwhile, in China, a minimum of 24% extra travel time is necessary for commuting during peak hours in major cities like Beijing, Shijiazhuang, and Chongqing [2]. Curbing vehicle purchases is not a feasible solution, and expanding traffic infrastructure is costly. There is a pressing need to mitigate pollution emissions from traffic congestion by leveraging technological innovations to improve environmental conditions, while enhancing the efficiency traffic signal systems appears to be a more manageable approach [3].

Currently, many intersections operate on a fixed-time traffic signal system that sets the timing of traffic signals based on historical data rather than in response to real-time traffic needs. Conventional traffic signal control techniques typically use predefined rules derived from strategies based on expert experience. These methods lack the capability to dynamically adjust signal phases based on instantaneous traffic flow data. In the context, Adaptive Traffic Signal Control (ATSC) [4,5,6] stands out. ATSC can be used to optimize traffic flow across regional road networks and effectively reduces congestion [7] by dynamically altering signal phases in response to current traffic conditions. The concept of ATSC was introduced many years ago, and key systems such as the Split Cycle Offset Optimization Technique (SCOOT) [8] and Sydney Coordinated Adaptive Traffic System (SCATS) [9] are other notable examples.

With the explosive growth of Deep Learning (DL), an increasing array of DL-based ATSC methodologies are emerging. Sun et al. [10] employed Convolutional Neural Networks (CNNs) to analyze image data from traffic cameras, deriving a method for predicting short-term traffic flow and thereby enabling real-time signal optimization. Similarly, Kong et al. [11] utilized Recurrent Neural Networks (RNNs) to process historical traffic data, facilitating proactive signal adjustments. In addition, traffic signal control is a sequential decision-making process that can be formulated as a Markov Decision Process (MDP). Thus, it is possible to frame this issue as a problem that can be solved using reinforcement learning (RL) [12,13,14]. One of the earliest contributions of RL to ATSC is based on *Q*-learning [15], and there are some other works applying RL for ATSC in intersection environments [16,17,18], where the focus is on dynamically adjusting traffic signals based on real-time and historical data to enhance the overall efficiency of traffic management systems.

Recently, RL has been enhanced using deep neural networks, giving rise to a subfield known as deep reinforcement learning (DRL). DRL combines the advantages of powerful hierarchical feature extraction and nonlinear approximation abilities, with the interaction between the agent and the environment. This integration has a range of appealing attributes, generating significant academic interest for the application of DRL in ATSC [7,19,20]. Li et al. [21] employed stacked autoencoders in RL for ATSC to ascertain the *Q*-function, facilitating the efficient compression and storage of agent inputs. Wei et al. [22] contributed an advancement to the field by integrating reward and policy interpretation into a DQN-embedded ATSC system. Nishi et al. [23] modeled the impact of neighboring nodes using a static adjacency matrix within the framework of GCN. Additionally, Wu et al. [24] proposed using CNNs for the improved extraction of state information features relevant to network topology-related issues, specifically addressing the ATSC problem. Wang et al. [25] used a decentralized RL approach with a region-aware strategy, incorporating an actor–critic model and graph attention networks to optimize traffic signal control. Zheng et al. [26] introduced the FRAP model, which optimizes traffic signal control by leveraging phase competition and symmetry invariance. Furthermore, Zhang et al. [27] applied meta-reinforcement learning using model-agnostic meta-learning (MAML) and flow clustering to generalize traffic signal control across diverse environments with support from a WGAN-generated traffic flow dataset. DRL excels over traditional RL by efficiently handling high-dimensional, complex data and automatically extracting nonlinear features while additionally surpassing DL by integrating decision-making and control capabilities, thereby enabling continuous learning and an effective balance between exploration and exploitation in dynamic environments.

However, due to the rapidly changing traffic conditions and the high dimensionality of traffic information, current DRL-based ATSC algorithms struggle to effectively capture the relationships between traffic information across different time sequences and to adequately understand traffic scenarios. The primary deficiencies are manifested as follows. Firstly, their reasoning capabilities are insufficient, resulting in non-stationarity during the training process. Secondly, most ATSC algorithms require extensive data to learn the optimal policy, necessitating greater computational resources and time requirements [28]. In addition, traditional DRL agents are specialized in their training environment, limiting their generalization and transferability to new environments. These considerations restrict the application of DRL to simple traffic scenarios, hindering its applicability in complex real-world traffic situations.

To address these issues, a novel DRL-based ATSC approach named Sequence Decision Transformer (SDT) is proposed, in which DRL is formulated as a sequence decision model. Given that large language models (LLMs) like the GPT series and BERT have demonstrated outstanding performance in natural language processing, computer vision, and reinforcement learning [29], the robust understanding and reasoning capabilities of LLMs are applied to the ATSC problem to accommodate the high dynamics of traffic conditions and the high dimensionality of traffic information. This enables SDT to incrementally learn optimal policy within complex traffic flows. The algorithm employs an encoder–decoder structure, stores historical trajectories in the replay buffer, and utilizes Proximal Policy Optimization (PPO) to update parameters, effectively addressing challenges in ATSC such as the demand for large amounts of data and the low generalization performance.

The main contributions of this paper are summarized as follows:

(1) The ATSC problem is first converted into a DRL formulation using a Markov Decision Process (MDP), where the essential elements, such as the observation space, action space, and reward functions, are defined with the goal of improving traffic efficiency and reducing congestion. This article introduce SDT, an extension of a standard MDP, with an encoder–decoder structure. This framework serves as means for deriving a formulation from the ATSC problem.

(2) Secondly, the SDT is employed to solve the ATSC problem formulated as an MDP. SDT utilizes a transformer to process data in parallel, which can potentially reduce training time and accommodate larger observation and action spaces. The self-attention mechanism in SDT captures dynamic changes in the environment and enhances the representation of general features, addressing issues related to non-stationarity and improving model generalization in new environments. Additionally, PPO is introduced, using a truncated probability ratio to limit the policy update step, thereby preventing large policy changes during training and increasing the stability of the training process.

(3) Thirdly, extensive experiments were carried out across various traffic scenarios. The experimental results demonstrate that the presented ATSC method based on SDT consistently excels at alleviating traffic congestion and enhancing the efficiency of the traffic system. The SDT model, compared to PPO, a DQN for ATSC, and FRAP, shows improvements of 26.8%, 150%, and 21.7% over traditional ATSC algorithms, and 18%, 30%, and 15.6% over the state-of-the-art (SOTA) under the most complex conditions.

The rest of this paper is organized as follows: The ATSC problem and the ATSC framework based on SDT are introduced and defined in Section 2. In Section 3, the ATSC problem is formulated as a Markov decision process. Section 4 details the algorithm, including its structure and update policy. The experiments are presented in Section 5, followed by the conclusions in Section 6. Appendix A details the explanation of notations appeared in this article.

## 2. Problem Statement

### 2.1. Problem Definition

**Figure 1:**
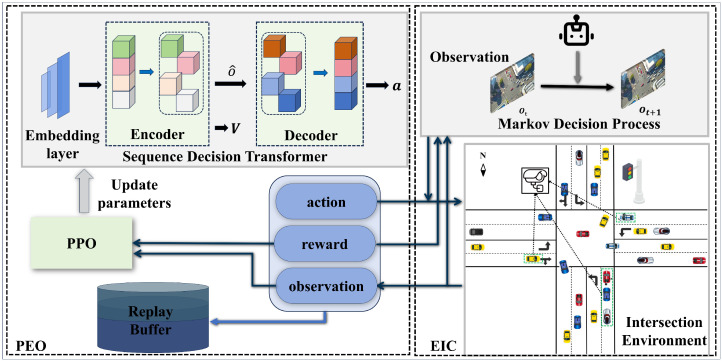
Conceptual framework of sequence decision transformer.

The intersection scenario controlled by an ATSC agent is illustrated in Figure 1. This represents a classic multiple-lane cross intersection, with multiple incoming lanes and outgoing lanes in each direction. When vehicles approach the intersection, they are presented with three options: go straight, turn left, or turn right. All vehicles’ passing choices directly affect future traffic conditions. The ATSC agent focuses on traffic flow information that affects traffic efficiency to make and control the traffic signal, specifically the traffic flow information from the incoming lanes.

The ATSC agent periodically obtains information for each lane $i(i\leq N_{lane}$ and $\left.i\in N^{+}\right)$:

(1) $$s_{i}=\left[\begin{array}{c} n_{i}\\ v_{i} \end{array}\right]$$

where $N_{lane}$ is the maximum number of lanes, $n_{i}\in R$ is the number of vehicles belonging to lanes *i* at step t∈N, and $v_{i}\in R$ is the average velocity of vehicles in lane *i* at step *t*.

ATSC methods are typically embedded within traffic signal control devices, such as traffic lights, in transportation systems. In regular time steps, the ATSC agent, embedded with the SDT algorithm, will determine the traffic signal phase *p* from $A=\{phase_{1},\ldots ,phase_{N}\}$ based on real-time traffic data gathered from external equipment like sensors and cameras across all lanes. The selected phase controls the intersection, and *A* denotes the set of traffic signal phases. Furthermore, the ATSC methods embedded in the traffic system are adjustable, allowing relevant personnel to modify the adjustable parameters of the ATSC methods to achieve superior operational efficiency.

### 2.2. Methodological Framework

As shown in Figure 1, the entire framework of SDT is depicted. It utilizes a transformer neural network architecture that includes an encoder and a decoder, forming an actor–critic structure. The encoder is tasked with receiving observations, learning the representations of these observations, and inputting the encoded observations into the decoder. As part of its role as a value network, the encoder also outputs the value of the received observations, which is utilized for the subsequent update of its parameters. The decoder, working as the policy network, is responsible for receiving the encoded data from the dncoder and outputting the action that the agent is required to execute. In addition, PPO is responsible for updating the parameters of the policy network using data from historical trajectories, enabling the ATSC agent to make better decisions.

The framework based on SDT-based ATSC is comprised of two parts: Environment Interaction Collecting (EIC) and Policy Evaluation Optimization (PEO). The EIC is tasked with acquiring updated neural network parameters, then employing these parameters to collect data from a road intersection environment. This component employs MDP to formally express the process through which an ATSC agent collects traffic information, executes actions, and receives rewards within an intersection environment. This procedure subsequently produces discrete time-series trajectory data, encompassing observations, actions, and rewards. These data form the basis for optimizing the neural network. The PEO works in conjunction with the EIC, using the data gathered from the EIC to update the SDT neural network. This component features a PPO method that solves the MDP problem to update the policy for maximizing ATSC performance. It then aligns the updated parameters with the EIS for subsequent rounds of collecting and optimization in a repetitive cycle, continuing until the targeted traffic performance at the intersection is achieved.

## 3. Representation of ATSC Problem to Markov Decision Process

### 3.1. Markov Decision Process

The Markov decision process (MDP), widely recognized for formally expressing the process through which a agent traverse the environment, fundamentally falls under the category of a discrete-time decision-making architecture. An MDP is defined by a tuple $\left(O,A,P,R,T\right)$, where:

- *O* is the observation space of the agent, where observations are the pieces of information that an agent receives $o_{t}\in O$ at each time step t∈N.
- *A* represents the set of action space of the agent in the environment, where the agent performs $a_{t}\in A$ in the discrete time step *t*.
- $R:O\times A\to \left[-R_{\max},R_{\max}\right]$ is a function that gives rewards; this function maps observation o to a numerical reward.
- P:O×A×O→R represents the transition probability function, which defines the dynamics of the environment.
- T:O×A→O is a function that describes the probability of transitioning from observation $o_{t}$ to observation $o_{t+1}$, given a particular action $a_{t}$. $T\left(o,a,o^{\prime }\right)=P\left(o_{t+1}=o^{\prime }|o_{t}=o,a_{t}=a\right)$.

Following the MDP model, the agent interacts with the environment in discrete time steps. At time step *t*, the agent produces an observation $o_{t}$ by interacting with the environment, and the agent performs an action $a_{t}$. After the agent has taken the action, it receives a reward $R\left(o_{t}|a_{t}\right)$. The environment then transits to a new observation $o_{t+1}$ based on the probability function $P\left(o_{t+1}|o_{t},a_{t}\right)$. When the trajectory $\tau =\left(o_{0},a_{0},o_{1},\ldots \right)$ from an epoch is gathered, the policy π undergoes an update, subsequently enabling the agent to continue interacting with the environment using this newly updated policy. The goal of an MDP is to enable the agent to learn the optimal policy $\pi^{*}$ that maximizes the total accumulated reward over time through continuous policy updates.

### 3.2. Converting the ATSC Problem to MDP

In this section, the ATSC problem is transformed into an MDP problem by defining its basic elements, including the observation space, action space, and reward function set. Table 1 shows the observation and action space in this work.

**Observation:** The observation space is used to describe the observation information during the intersection between the agent and the environment. A reasonable observation space is crucial for efficient training of DRL algorithm. In the ATSC system, the capacity to accurately extract and reconstruct observation information from the complex and dynamic environment of intersections is significant, as it determines the ability to output appropriate actions accurately.

The observation space is defined as the sum of the observation of lanes in the current scenario, i.e., $O=\sum_{i=1}^{N_{l}ane}O^{i}$. Considering that the traffic conditions at intersections vary according to the dynamic and static traffic information of each lane, the observation subset is set as $O^{i}=\left[n_{i},t_{i},q_{i},v_{i}\right]$, where $n_{i}$ represents the total number of vehicles in lane *i* at time *t*, $t_{i}=\frac{1}{n_{i}}\sum_{m=1}^{n_{i}}\left\{\begin{array}{c} t_{m},v_{m}<0.1\;m/s\\ 0,\;v_{m}\geq 0.1\;m/s \end{array}\right.$ represents the average waiting time of vehicles with a velocity of less than 0.1m/s in lane *i* at time *t*; $v_{m}$ and $t_{m}$ are the velocity and the waiting time of vehicle *m*, respectively; $q_{i}=\sum_{m=1}^{n_{i}}\left\{\begin{array}{c} 1,v_{m}<0.1\;m/s\\ 0,v_{m}\geq 0.1\;m/s \end{array}\right.$ represents the queue length of lane *i* at time *t*, which can be denoted by the number of queued vehicles; and $v_{i}=\frac{1}{n_{i}}\sum_{m=1}^{n_{i}}\frac{v_{m}}{v_{\max}^{\prime }}$ represents the average velocity of vehicles in lane *i* at time *t*. The above observation space can accommodate complex and randomly changing traffic intersection scenarios, effectively characterizing the traffic conditions at the intersection.

**Action:** In this paper, the discrete set of traffic light phases is considered as the action space $A=\{{\mathrm{phase}}_{1},\ldots ,{\mathrm{phase}}_{N}\}$ for the agent. Upon receiving an observation $o_{t}$ at time step *t*, the agent selects and executes an appropriate action *p* from $\left\{0,\ldots ,N_{pha}-1\right\}$, and $N_{pha}$ indicates the quantity of phases. By executing *p*, the agent determines the roads that are allowed and disallowed to pass at time t+1 and maintains the phase $phase_{j}$ duration.

**Reward:** The reward function serves as quantitative feedback received by the agent from the environment after executing an action. In this study, the reward function guides the agent in continuously exploring and learning optimal strategies while maximizing rewards. The design of these functions is crucial for the convergence speed of DRL algorithms. If the reward function is overly optimized for a specific scenario, it may lead to overfitting, reducing the trained model’s ability to adapt to new scenarios. Generally, performance metrics should be designed based on the actual objectives intended to be achieved in the environment. To comprehensively describe the environment, the reward function focuses on holistically enhancing the traffic efficiency at intersections, ensuring that the reward system accurately reflects the desired outcomes in the environment, not just how the agent should behave. Such a design helps avoid overtraining in specific environments while enhancing the model’s adaptability and generality in new scenarios. Therefore, considering all aspects, the reward function comprises the following components:

(1) The total number of vehicles ($R_{num}$): By focusing on the total number of vehicles, the reward function can directly impact and assess traffic flow at intersections. Reducing the total number of vehicles at intersections aids in alleviating traffic congestion and facilitating smoother traffic. This metric effectively reflects the intersection’s capacity and efficiency in managing traffic flow.

(2) $$R_{num}=\sum_{i=1}^{N_{lane}}n_{i}$$

(2) The average speed of vehicles ($R_{vol}$): Average speed is one of the key metrics used to assess traffic efficiency. By increasing the average speed of vehicles, traffic congestion can be reduced and travel times shortened, thereby enhancing the overall efficiency of the intersection. Higher average speeds generally indicate good traffic conditions, where vehicles do not need to frequently stop or slow down.

(3) $$R_{vol}=\frac{1}{N_{lane}}\sum_{i=1}^{N_{lane}}v_{i}$$

(3) The average queue lengths of all vehicles ($R_{que}$): This metric reflects the queuing situation of vehicles at the intersection. Shorter queue lengths mean that vehicles can pass through the intersection more quickly, reducing waiting and idling times, which helps to enhance the continuity of traffic flow and reduce congestion. Controlling queue length can effectively optimize the distribution of traffic flow and the scheduling of traffic signals at the intersection.

(4) $$R_{que}=\frac{1}{N_{lane}}\sum_{i=1}^{N_{lane}}q_{i}$$

(4) The average waiting time of all vehicles ($R_{time}$): The average waiting time is an important metric for measuring the efficiency of an intersection. By reducing the average waiting time of vehicles at the intersection, the smoothness and efficiency of traffic flow can be significantly improved.

(5) $$R_{time}=\frac{1}{N_{lane}}\sum_{i=1}^{N_{lane}}t_{i}$$

In conclusion, the reward function is formulated as follows:

(6) $$R=w_{1}\times R_{num}+w_{2}\times R_{vol}+w_{3}\times R_{que}+w_{4}\times R_{time}$$

Here, $w_{1},w_{2},w_{3},w_{4}$ represent the weights for the items of the reward function, respectively. The final goal of the proposed algorithm is to optimize a weighted sum of four objectives, which serves as a normalization method. This weighted summation approach was chosen because the scales of these four factors vary significantly in the initial episodes for each environment. Simply adding them as a reward would cause the agent to focus disproportionately on the factors with larger magnitudes, potentially leading to the algorithm’s failure to converge.

Weighting these four metrics ensures that each indicator has an appropriate proportion within the reward function, thereby overcoming issues of differing scales and uneven impact among the indicators. This design helps the algorithm balance the importance of different metrics, avoiding the overoptimization of a single metric while neglecting others that are equally important. Additionally, this weighted approach enhances the stability and convergence of the algorithm, enabling it to effectively learn and adapt in various traffic environments, ultimately achieving the goals of reducing congestion and improving traffic efficiency.

## 4. Sequence Decision Transformer

This section provides a detailed description of the SDT algorithm used to solve the MDP problem; an advanced framework using an encoder–decoder architecture. The encoder processes environmental observations and evaluates their values, while the decoder determines actions based on the policy updated through PPO to enhance decision-making accuracy and adaptability in dynamic traffic conditions.

### 4.1. Structure and Update

**Figure 2:**
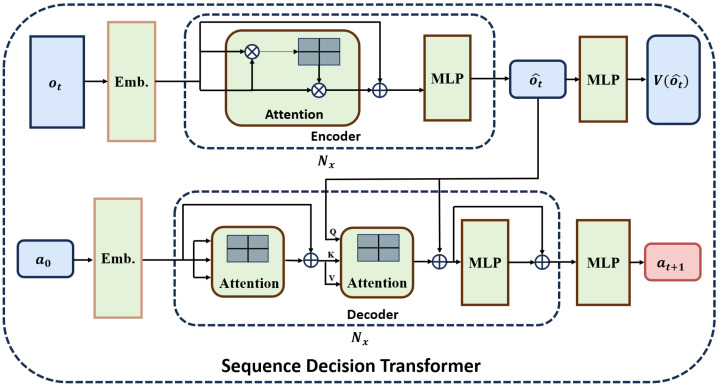
The encoder–decoder architecture of SDT. At each time step, the encoder takes in the agent’s observations and encodes them into a sequence of latent representations, which is then passed into the decoder, and the decoder generates the agent’s optimal action.

Figure 2 depicts the conceptual structure of SDT, which is mainly comprised of two components: the encoder and the decoder. The encoder is responsible for receiving observations $o_{t}$ from the environment, learning representations of observations ${\hat{o}}_{t}$, and outputting the value $V_{t}$ of the received observations at time *t*. The decoder is in charge of outputting the action $a_{t}$ to be executed by the agent at time *t*. Consequently, after the agent performs a predetermined number of steps, a portion of the historical trajectory τ will be given to PPO for updating the policy π and the encoder–decoder networks.

The transformer model is the key to SDT, specifically the self-attention mechanism, which computes the response at a position in a sequence by attending to all positions and drawing global dependencies between the input and output. The attention function is written as follows:

(7) $$Attention\left(Q,K,V\right)=soft\max\left(\frac{QK^{T}}{\sqrt{d_{k}}}\right)V$$

(8) $$Q=S\times W^{q},K=S\times W^{k},V=S\times W^{v}$$

where *S* denotes the input sequence, and $W^{q},W^{k},W^{v}$ are the weight matrix. *Q*, *K*, and *V* correspond to the vector of queries, keys, and values that can be learned during training, and $\sqrt{d_{k}}$ represents the dimension of *Q* and *K*. Self-attention refers to cases when Q,K,V share the same set of parameters.

**Encoder:** The function of the encoder, whose parameters are represented by ϕ, is as follows: Observation $o_{t}$ first passes through an embedding layer $o_{E}=Emb\left(o_{t}\right)$, which maps discrete input into vectors of continuous numbers in a lower-dimensional space to enhance computational efficiency. It then passes through the self-attention mechanism and residual connections $o_{A}=Attention\left(Q^{o_{E}},K^{o_{E}},V^{o_{E}}\right)+o_{E}$ to prevent gradient vanishing and network degradation. Finally, the data pass through a multi-layer perception (MLP) to obtain the ${\hat{o}}_{t}=MLP\left(o_{A}\right)$, which is an input of the decoder. In addition, ${\hat{o}}_{t}$ is also utilized to calculate the value $V\left({\hat{o}}_{t}\right)=MLP\left({\hat{o}}_{t}\right)$, facilitating subsequent updates within the network. The role of the encoder is similar to that of a critic, as it aims to approximate the value function. Its objective is to minimize the empirical Bellman error:

(9) $$L_{Encoder}\left(\phi \right)=\frac{1}{T}\sum_{t=0}^{T-1}{\left[R\left(o_{t},a_{t}\right)+\gamma V_{\hat{\phi }}\left({\hat{o}}_{t+1}\right)-V_{\phi }\left({\hat{o}}_{t}\right)\right]}^{2}$$

where $\hat{\phi }$ represents the target network’s parameters, which are non-differentiable and updated every few time steps.

**Decoder:** The embedded action $a_{0}$ (an arbitrary symbol indicating the start of decoding) passes into the decoder, whose parameters are denoted by θ. The decoder consists of many decoder blocks, like the encoder. Each block contains two attention mechanisms and one MLP. The first mechanism is responsible for receiving $a_{0}$ and generating $a_{A1}=Attention\left(Q^{a_{E}},K^{a_{E}},V^{a_{E}}\right)+a_{E}$, where $a_{E}$ is obtained by passing through an embedding layer that performs a dimensional transformation. The attention scores are calculated in the second attention mechanism $a_{A2}=Attention\left(Q^{{\hat{o}}_{t}},K^{a_{A1}},V^{a_{A1}}\right)+{\hat{o}}_{t}$. After passing through two MLP, the action representation $a_{t+1}=MLP\left(MLP\left(a_{A2}\right)+a_{A2}\right)$ is output. The second MLP outputs the probability distribution of the agent’s actions, namely the policy $\pi_{\theta }\left(a_{t+1}|{\hat{o}}_{t},a_{t}\right)$.

Then, PPO is utilized to update θ. PPO is a policy gradient method for reinforcement learning that balances implementation simplicity with sample efficiency. It addresses the limitations of previous strategies by allowing for multiple epochs of minibatch updates while avoiding excessively large policy updates that could destabilize training. This stability is achieved through a clipped objective function, which restricts the update step to remain within a small range around the old policy. The clipped objective function is as follows:

(10) $$L^{CLIP}\left(\theta \right)=E_{t}\left[\min\left(r_{t}\left(\theta \right){\hat{A}}_{t},clip\left(r_{t}\left(\theta \right),1\pm \epsilon \right){\hat{A}}_{t}\right)\right]$$

Here, $r_{t}\left(\;\cdot \;\right)$ is the policy ratio, and ϵ is a small positive number used to define the clipping range. ${\hat{A}}_{t}$ is an estimate of the advantage function, which is represented as follows:

(11) $$A\left(o,a\right)=Q\left(o,a\right)-V\left(o\right)$$

(12) $$Q\left(o,a\right)=E_{o_{1:\infty }∼P,a_{1:\infty }∼\pi }\left[R^{\gamma }|o_{0}=o,a_{0}=a\right]$$

(13) $$V\left(o\right)=E_{a_{0}∼\pi ,o_{1:\infty }∼P,a_{1:\infty }∼\pi }\left[R^{\gamma }|o_{0}=o\right]$$

It measures the relative benefit of choosing a particular action *a* in an observation *o* over the average action under the current policy. This function highlights the excess expected return of an action compared to the average policy performance, thereby facilitating more informed and effective policy updates and decision-making in learning algorithms.

Based on these theorems, the following clipped PPO objective is minimized to train the decoder:

(14) $$L_{Decoder}\left(\theta \right)=-\frac{1}{T}\sum_{t=0}^{T-1}\min\left(r_{t}\left(\theta \right){\hat{A}}_{t},clip\left(r_{t}\left(\theta \right),1\pm \epsilon \right){\hat{A}}_{t}\right)$$

(15) $$r_{t}\left(\theta \right)=\frac{\pi_{\theta }\left(a_{t+1}|{\hat{o}}_{t},a_{t}\right)}{\pi_{\theta_{old}}\left(a_{t+1}|{\hat{o}}_{t},a_{t}\right)}$$

### 4.2. Algorithm Overview

This section introduces the ATSC algorithm based on SDT, as presented in Algorithm 1. The SDT algorithm initiates by initializing the encoder and decoder networks parameters ϕ and θ, alongside a replay buffer *D* to store transitions (line 1). The environment is then reset for a initial observation $o_{0}$, $a_{0}$, $R_{0}$, etc., in the EIC (line 2). Within a loop that persists for a preset number of episodes, the algorithm, at each timestep *t* within an episode, calculates the value of $V_{t}$, subsequently selecting the most suitable action $a_{t}$. Following the execution of an action, the agent observes the outcome and receives a corresponding reward $R_{t}$, which, along with the value and the new observation $o_{t+1}$, is stored in the replay buffer (line 3–8). Upon the completion of each episode, the update of the encoder’s parameters and the decoder’ parameters is triggered. This update utilizes the computed returns derived from the accumulated data, specifically through the value function and PPO policy, as detailed in Equation (9) and Equation (14), respectively. This updating mechanism is systematically repeated at the end of each episode (line 9), ensuring continuous refinement and optimization of the network parameters based on the most recent episodic experiences. This iterative approach is integral to the model’s learning process, promoting enhanced performance and stability. This cyclical process aims to refine the agent’s policy for traffic signal timing, enhancing the management of intersection traffic flow.
**Algorithm 1:** Sequence Decision Transformer1:Initialization: Encoder network ϕ, Decoder network θ, and replay buffer *D*2:Reset environment and get initial observation $o_{0}$, action $a_{0}$, reward $R_{0}$ and current policy etc. in the EIC3:**for** episode = 1 to MAX_EPISODES **do**4:  **for** t = 0 to EPISODE_LENGTH **do**5:    Calculate value $V_{t}$ and select action $a_{t}$ from Encoder-Decoder6:    Obtain observation $o_{t+1}$ and reward $R_{t}$ after agent takes $a_{t}$7:    Store transition $\left(o_{t},V_{t},o_{t+1},R_{t}\right)$ in *D*8:  **end for**9:  Compute return and update parameters ϕ and θ every episode by
    $L_{Encoder}\left(\phi \right)=\frac{1}{T}\sum_{t=0}^{T-1}{\left[R\left(o_{t},a_{t}\right)+\gamma V_{\hat{\phi }}\left({\hat{o}}_{t+1}\right)-V_{\phi }\left({\hat{o}}_{t}\right)\right]}^{2}$ and
    $L_{Decoder}\left(\theta \right)=-\frac{1}{T}\sum_{t=0}^{T-1}\min(r_{t}\left(\theta \right){\hat{A}}_{t},clip\left(r_{t}\left(\theta \right),1\pm \epsilon \right){\hat{A}}_{t})$
10:**end for**


## 5. Experiment

To evaluate the proposed ATSC algorithm based on the Sequence Decision Transformer (SDT), two sets of experiments were conducted. All experiments were performed using Simulation of Urban Mobility (SUMO) [30], a traffic simulation tool. In the experiments, all intersections were set in a synthetic traffic environment. To ensure the practicality of the proposed model, the experiments focused on standard intersections, the most common type of traffic scenario in modern cities, where vehicles can proceed straight, turn left, or turn right. U-turns were not permitted in the designed synthetic scenarios, as most urban intersections do not allow direct U-turns due to the increased risk of traffic accidents. Therefore, the option for vehicles to make U-turns was excluded from our experiments.

Specifically, the first experiment demonstrates the training process of the proposed algorithm, showing the evolution of performance during iterations of policy updates and the final performance of the policy. The second experiment compares the performance of a fixed-time traffic signal, a baseline PPO method, a DQN algorithm specifically designed for ATSC problems [31], and a FRAP [26] algorithm to the SDT algorithm under various traffic scenarios. This section begins with the experimental setup and implementation details, followed by an analysis of the training and test results obtained.

### 5.1. Experiment Setting

The training scenario is a intersection that has 32 lanes, while each direction has 3 straight lanes, 1 right-hand lane, and 1 left-hand, lane and they are all 750 m long. The traffic flow density is approximately 600–5000 veh/h during the training process.

In SUMO, the shortest simulation time interval, namely, the time-step τ, is 1 s. The traffic flow, which is randomly generated at various moments within 3600 s, will enter incoming lanes, thereby pass by the intersection. The SDT algorithm has been trained 3 million times with an episode length of 500, which means that there are 6000 episodes in this training scenario. In the synthetic scenario, the weights assigned to each item of the discount reward function are {$w_{1},w_{2},w_{3},w_{4}$} = {0.5,−0.5,−2,−0.5}. Each decision-making *T* has an interval of 6 s, meaning the agent will decide whether to maintain the current phase or switch to another phase every 6 s. If *T* is set too short, such as 1 s, it can cause traffic lights to switch too often, which is impractical and increases the computational load. Conversely, if *T* is set too long, it will slow the agent’s learning rate. The parameters of the baseline PPO, DQN, and FRAP algorithm are restored to the original settings. Please refer to Table 2 for the detailed parameters of the SUMO simulator, baseline PPO method, DQN, FRAP algorithm, and SDT algorithm.

### 5.2. Analysis of Performance during Training Process

**Figure 3:**
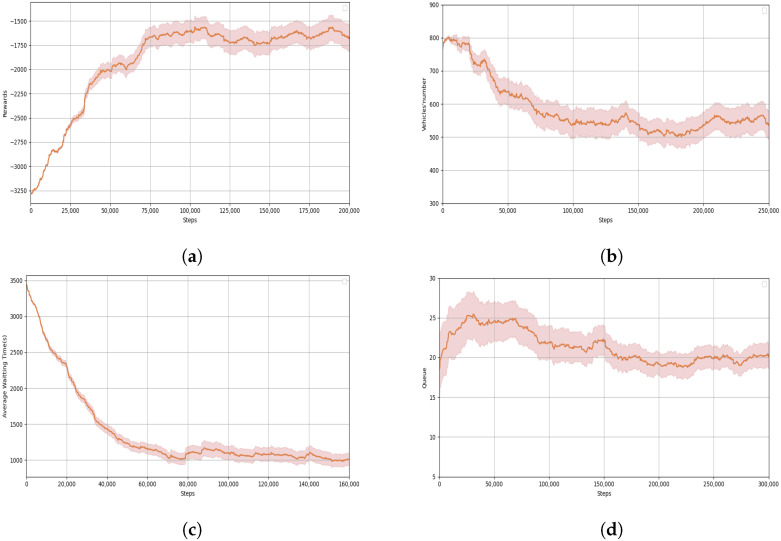
Performance of SDT during the training process. (**a**) Rewards curve with time steps. (**b**) Number of vehicles in the intersection over time. (**c**) Average waiting time for the intersection over time. (**d**) Queue length of the intersection over time.

In this experiment, a high-performing ATSC agent was successfully trained using the SDT algorithm. Figure 3a illustrates the changes in reward values throughout the training process. The curve in the graph represents the variation of the discount rewards as the number of time steps increases. During exploration of the agent and updating of the network parameters, the rewards curve gradually increases, indicating that the policy has become adept at efficiently handling problems, and the agent is continuously adapting to varying scenarios. At around 75,000 steps, the reward curve reaches its peak and subsequently fluctuates with minimal variance for an extended period. This suggests that the agent has learned the optimal policy after approximately 75,000 steps, and the subsequent fluctuations are due to randomness in traffic scenarios and vehicle flows at different times. The rapid convergence and superior policy performance can be attributed to the integration of the SDT framework, which leverages the encoder–decoder architecture for efficient data processing. The self-attention mechanism within the transformer model enables the SDT to capture intricate dependencies and dynamic changes in traffic conditions, thereby enhancing the learning process. Additionally, the application of PPO ensures stable and continuous policy updates, preventing large policy shifts and maintaining consistent performance improvements. This robust methodological framework contributes significantly to the observed rapid convergence and high-quality policy performance during training.

To validate the convergence of the rewards and each component, the changes in three reward components were recorded with respect to the number of steps. Figure 3b,c display the total number of vehicles and the average waiting time of vehicles, respectively. The trends in these two reward items are essentially consistent with the reward curve. The total number of vehicles decreases with the update of the policy, reaching a low point around 100,000 steps and subsequently fluctuating within a small range. The average waiting time reaches its lowest point after around 80,000 steps, as the policy updates and remains relatively stable, with only minor fluctuations thereafter. Figure 3d shows the queue length as the number of time steps increases. During the training process, the queue length initially increases slightly and then gradually decreases. As the policy is updated, the minimum queue length occurs around 160,000 steps and maintains relative stability thereafter with only minor fluctuations. Although the reward items converge at different times, the trends and the final outcomes are the same. This indicates that the convergence of the rewards is accompanied by the convergence of the reward items, and the traffic light at the intersection becomes more efficient in managing traffic flows under the influence of the SDT algorithm, leading to improved traffic conditions at the intersection.

### 5.3. Performance Comparison

**Figure 4:**
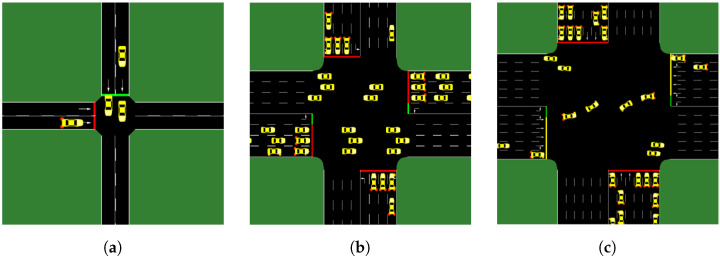
Performance comparison scenario of SDT, FRAP, baseline PPO, and FIXED method. (**a**) The easy scenario. (**b**) The medium scenario. (**c**) The hard scenario.

This section compares the performance of the policy trained using the SDT algorithm with the fixed-time method, a PPO method, a DQN tailored for the ATSC method, and a SOTA ATSC algorithm, FRAP. To demonstrate the performance of the SDT algorithm, three typical scenarios were used, which are illustrated in Figure 4. In all scenarios, traffic flow follows a Poisson distribution; namely, traffic flow is generated randomly, and the probability of arrival is the same within equal time intervals during the simulation period. The traffic density for each lane at the intersection remains constant and does not vary under different conditions such as peak hours.

- Easy scenario: In this scenario, traffic can only flow from north to south and from west to east, with each direction consisting of two lanes. The traffic density is 600 veh/h.
- Medium scenario: This scenario is similar to the training scenario, with each direction consisting of four lanes. The traffic density is 4000 veh/h.
- Hard scenario: This intersection has 48 lanes, while each direction has 4 straight lanes, 2 right-turn lane, and 2 left-turn lane. The traffic flow density is approximately 5000 veh/h.

In the scenarios described above, several experiments were also set up to compare these algorithms with the SDT algorithm:

- FIX6/12/18/24: The figures represent the fixed duration of each green light phase. In the FIXED experiments, the green light phases cycles with a fixed duration, simulating the traffic lights commonly found at most real-life intersections to influence the traffic conditions.
- Baseline PPO: This implementation is a standard, unmodified version of PPO without any specific design tailored for ATSC. The policy model is MLP, which is used to learn and optimize traffic signal control strategies from the traffic environment. However, due to the lack of specific design adjustments, this PPO algorithm represents a straightforward application in the ATSC problem.
- DQN [31]: This DQN-based DRL algorithm is designed for ATSC. It leverages a deep convolutional neural network (CNN) to extract useful features from traffic data and utilizes the Q-learning algorithm to identify the optimal traffic signal control strategy.
- FRAP [26]: FRAP models traffic signal control as a phase competition problem, giving priority to the traffic phase with the highest demand. By leveraging symmetry in traffic flow (flipping and rotation), FRAP reduces the problem’s complexity and state space, thus improving learning efficiency.

Table 3 shows final results of the comparison. To evaluate the performances of the different methods, three key metrics were used: the total number of vehicles at the intersection (vehicle number), the average speed of all vehicles (speed), and the average queue length at the intersection (queue). These metrics provide a straightforward reflection of the congestion level at the intersection, serving as core indicators for assessing the model’s performance, with their calculation formulas corresponding to Equations (2), (3), and (4), respectively. The results demonstrate that, across all scenarios, the SDT algorithm significantly outperforms the FIXED method and the baseline PPO method in all aspects.

In the easy scenario, SDT achieves improvements of 5% and 2% in vehicle number and speed compared to the FRAP method. These relatively modest improvements can be attributed to the lighter traffic conditions in this scenario, where the traditional ATSC algorithm is still sufficiently effective. However, the encoder–decoder architecture of SDT allows it to capture more nuanced dynamics of traffic flow, leading to slight performance gains.

In the medium scenario, the improvements by SDT become much more pronounced, with gains of 23%, 65%, and 13% in vehicle number, speed, and queue, respectively. This significant enhancement is primarily due to the self-attention mechanism within the SDT’s transformer model, which captures complex dependencies and dynamic interactions among traffic flows, enabling SDT to adjust signal control more precisely than the DQN and FRAP method. As traffic fluctuations and complexity increase in medium scenarios, FRAP’s limited capacity to model temporal dependencies and interactions among multiple streams becomes more apparent, leading to inferior performance.

In the hard scenario, SDT’s advantages are even more evident, with improvements of 26%, 24%, and 34% in vehicle number, speed, and queue, respectively. In such high-complexity scenarios, FRAP and the DQN struggle to cope with the high traffic volume and intricate interactions, resulting in significantly lower performances. In contrast, SDT not only leverages its encoder–decoder architecture to effectively process complex data but also benefits from the stable and continuous policy updates of the PPO algorithm, preventing large policy shifts and ensuring consistent performance improvements. SDT’s ability to dynamically adjust according to real-time traffic conditions greatly alleviates congestion and enhances throughput efficiency.

However, it is worth noting that the SDT algorithm relies heavily on computational resources and is sensitive to hyperparameter settings. While these factors contribute to its high performance, they also highlight areas for further optimization. Reducing computational demands and enhancing the algorithm’s robustness to hyperparameter variations could broaden SDT’s applicability.

Overall, as the scale of the intersection and traffic flow increases, the SDT algorithm, with its sophisticated architecture and advanced learning mechanisms, effectively addresses the limitations of traditional methods such as FRAP in terms of dynamic adaptability and handling complex traffic conditions, achieving more flexible and efficient traffic management.

## 6. Conclusions

The SDT introduced in this paper represents a significant advancement in the field of ATSC using DRL. By adopting a sequence decision-making framework for traffic control, the SDT model effectively addresses the high variability and complexity of urban traffic flows with remarkable efficiency. The integration of a PPO-based algorithm within this framework ensures that the model remains stable and adaptable across various traffic conditions, which is essential for real-world applications. Our comprehensive experimental evaluation demonstrates that the SDT outperforms existing methods, including fixed-time, the baseline PPO method, and advanced FRAP-based systems, by significantly improving traffic throughput and reducing congestion. These improvements are achieved without compromising the system’s adaptability and responsiveness to dynamic traffic patterns, highlighting the potential of DRL in transforming traffic management systems. The success of the SDT model in these experiments indicates that it can serve as a robust framework for future developments in intelligent transportation systems, potentially leading to more sustainable and efficient urban environments. However, further research is needed to address certain limitations. Firstly, the SDT algorithm requires extensive computational resources for training, which may limit its scalability. Secondly, the potential sensitivity to hyperparameter settings necessitates careful tuning to ensure optimal performance across different scenarios. To mitigate these issues, future work will focus on optimizing the computational efficiency and exploring automated hyperparameter tuning techniques, thereby enhancing the overall robustness and applicability of the SDT algorithm.

## Figures and Tables

**Table 1:** Observation space and action space.

Notation	Description	Range	Scale
$v_{i}$	Average velocity of lane *i*	$\left[0,V_{\max}\right]$ m/s	$\left[0,V_{\max}\right]$
$t_{i}$	Waiting time of lane *i*	[0,∞] s	[0,∞]
$q_{i}$	Queue length of lane *i*	[0,∞]	[0,∞]
$n_{i}$	Total number of vehicles in lane *i*	$\left[0,N_{\max}\right]$	$\left[0,N_{\max}\right]$
*p*	Traffic signal light phase	$\left\{phase_{1},\ldots ,phase_{N}\right\}$	$\left\{0,\ldots ,N_{pha}-1\right\}$

**Table 2:** Main parameters of the experiments.

Parameters	Values
**SUMO simulator**	
Time-step **τ**	1 s
Traffic flow	600–5000 veh/h
Edge length	750 m
**Baseline PPO**	
Learning rate α	1 × $10^{-3}$
Batch size B	64
Target update interval $\Delta t_{\mathrm{target}}$	1024
Policy model	MLP
Exploration rate ϵ	0.1
Discount factor γ	0.95
Exploration rate decay $\epsilon_{\mathrm{decay}}$	0.995
Minimum exploration rate $\epsilon_{\min}$	0.01
**DQN algorithm**	
Learning rate α	1 × $10^{-3}$
Replay buffer size N	50,000
Target update interval $\Delta t_{\mathrm{target}}$	500
Optimizer	Adam
Update strategy π	MLP
Exploration factor $\epsilon_{\mathrm{frac}}$	0.05
**FRAP algorithm**	
Number of layers	2
Dense dimension	20
Exploration rate ϵ	0.1
Exploration rate decay $\epsilon_{\mathrm{decay}}$	0.995
Minimum exploration rate $\epsilon_{\min}$	0.01
Demand shape	1
**SDT algorithm**	
Learning rate α	5 × $10^{-4}$
Discount factor γ	0.99
Decision-making T	6 s
PPO clip	0.05
Optimizer	Adam
Timesteps	3 × $10^{6}$
Episode length	500

**Table 3:** Performance comparison.

Scenarios	Metrics	FIX6	FIX12	FIX18	FIX24	PPO	DQN	FRAP	SDT
Easy	Vehicle number	3.4	3	3.7	3.4	4.1	1.9	2.0	**1.9**
	Average speed	0.73	0.63	0.46	0.39	0.59	0.88	0.85	**0.87**
	Queue length	0.125	0.2	0.4	0.47	0.15	0.025	0.025	**0.025**
Medium	Vehicle number	1434	1129.6	1055.5	1004.3	949.5	929.6	427.1	**326.3**
	Average speed	0.056	0.1	0.11	0.13	0.07	0.08	0.23	**0.38**
	Queue length	34.48	33.66	34.22	34.03	54.7	37.7	29.7	**25.6**
Hard	Vehicle number	352.8	301	280.9	426.7	629.1	143.7	154.5	**113.3**
	Average speed	0.092	0.166	0.129	0.1	0.120	0.095	0.136	**0.168**
	Queue length	6.35	4.56	5.15	7.15	34.01	4.76	4.82	**3.18**

## Data Availability

The data presented in this study are available from the corresponding author upon request.

## Appendix A

The appendix contains a comprehensive table that lists and explains the notations used throughout the main text, providing a clear and concise reference for better understanding of the content discussed.

**Table A1:** List of notations.

Notation	Description
$s_{i}$	The raw information of lane *i*
$N_{lane}$	The maximum number of lanes
*O*	The observation space
*A*	The action space
*R*	The reward function
*P*	The transition probability function
*T*	The probability of transitioning from observation $o_{t}$ to $o_{t+1}$
$O^{i}$	The observation space of lane *i*
$R_{num},R_{vol},R_{que},R_{time}$	The items of the reward function
$w_{1},w_{2},w_{3},w_{4}$	The weights for items of the reward function
$W^{q},W^{k},w^{v}$	The attention weight matrix
Q,K,V	The vector of queries, keys, and values
$\sqrt{d_{k}}$	The dimension of *Q* and *K*
L(·)	The loss function
Q(o,a)	The Q function
V(o)	The value function
A(o,a)	The advantage function
